# Interaction of ncRNA and Epigenetic Modifications in Gastric Cancer: Focus on Histone Modification

**DOI:** 10.3389/fonc.2021.822745

**Published:** 2022-01-26

**Authors:** Qingfan Yang, Yu Chen, Rui Guo, Yalan Dai, Liyao Tang, Yueshui Zhao, Xu Wu, Mingxing Li, Fukuan Du, Jing Shen, Tao Yi, Zhangang Xiao, Qinglian Wen

**Affiliations:** ^1^ Department of Oncology, The Affiliated Hospital of Southwest Medical University, Southwest Medical University, Luzhou, China; ^2^ South Sichuan Institute of Translational Medicine, Luzhou, China; ^3^ Laboratory of Molecular Pharmacology, Department of Pharmacology, School of Pharmacy, Southwest Medical University, Luzhou, China; ^4^ Cell Therapy & Cell Drugs of Luzhou Key Laboratory, Luzhou, China; ^5^ School of Chinese Medicine, Hong Kong Baptist University, Hong Kong, Hong Kong SAR, China

**Keywords:** ncRNA, histone modification, gastric cancer, epigenetic modifications, mechanisms

## Abstract

Gastric cancer has developed as a very common gastrointestinal tumors, with recent effective advancements in the diagnosis and treatment of early gastric cancer. However, the prognosis for gastric cancer remains poor. As a result, there is in sore need of better understanding the mechanisms of gastric cancer development and progression to improve existing diagnostic and treatment options. In recent years, epigenetics has been recognized as an important contributor on tumor progression. Epigenetic changes in cancer include chromatin remodeling, DNA methylation and histone modifications. An increasing number of studies demonstrated that noncoding RNAs (ncRNAs) are associated with epigenetic changes in gastric cancer. Herein, we describe the molecular interactions of histone modifications and ncRNAs in epigenetics. We focus on ncRNA-mediated histone modifications of gene expression associated with tumorigenesis and progression in gastric cancer. This molecular mechanism will contribute to our deeper understanding of gastric carcinogenesis and progression, thus providing innovations in gastric cancer diagnosis and treatment strategies.

## 1 Introduction

Gastric cancer (GC) is ranking the fifth most common cancer in the world, with over one million cases in 2018, nearly two-thirds of which occur in developing countries. GC remains the third leading cause of tumor-related death, just behind lung cancers and colorectal cancers (1–3). GC is a highly heterogeneous disease, and according to Lauren’s classification, it can be classified into two histological subtypes: the intestinal type and the diffuse type. High-throughput technologies have been well developed, and a new molecular classification is proposed by The Cancer Genome Atlas (TCGA), which subdivided GC into chromosomal instability (CIN)、genomic stability (GS)、microsatellite instability (MSI) and Epstein-Barr-Virus positivity (EBV) (4). There are four subtypes according to the WHO classification system, including papillary, tubular, signet ring, and mucinous (5).

GC is a heterogeneous cancer with multifactorial and unique epigenetic and genetic events, but the pathogenesis and molecular mechanisms remain elusive (6, 7). Both genetic and environmental factors are able to participate in the development and progression of GC (8). Genetic mutations have been considered as driving force for cancer development. However, current research data show substantial evidence that epigenetic alterations play an essential role in the development and progression of cancers, including GC (9, 10). Epigenetics refers to alterations in the process of gene expression without changes in DNA sequence, which are heritable, transient and reversible in gene expression (11). Epigenetic changes in cancer include DNA methylation, histone modifications and chromatin remodeling. The occurrence as well as progression of GC can be partially ascribed to environmental factors, including age, high salt intake, a diet low in fruits/vegetables and H. pylori infection (1).. However, environmental effects on GC are controllable, and only by exploring epigenetic regulation in depth can epigenetic alterations be controlled and managed. Histone modification occupies an important place in the regulatory mechanisms of epigenetics, and it is detected mainly in amino acids and carbohydrates, which also plays an essential role in cancer development (12). Recently, a large amount of literature indicates that noncoding RNAs (ncRNAs) are closely related to histone modifications in GC. In this review, the epigenetic alterations of histone post-transcriptional modifications associated with ncRNAs in gastric cancer have been intensively discussed.

## 2 A Brief Description of Histone Post-Transcriptional Modifications

Chromatin is a linearly arranged structure composed of millions of nucleosomes consisting mainly of DNA, RNA and histones (13). The fundamental unit of human genetic material is the nucleosome and consists of a histone octamer wrapped by a double-stranded DNA, this octamer contains two copies of histones H2A, H2B, H3 and H4 (14). Post-translational modifications of histones, including multiple covalent modifications such as methylation, acetylation, phosphorylation, ubiquitination, sumoylation, etc. They largely regulate DNA accessibility and gene expression (15). Currently, aberrant regulation of histone modifications has now been investigated in many kinds of cancers, and the role of histone modifications is being extensively studied. In this paper, we briefly describe the various types of histone post-transcriptional modifications and focus on the interaction between ncRNAs and histone acetylation/methylation in GC.

### 2.1 The Role of Histone Acetylation in Cancer

Histone acetylation, a reversible and dynamic process, is inversely regulated by deacetylases (HDACs) and histone acetyltransferases (HATs) (16). Acetylation neutralizes the positively charged lysine side chains to make the DNA structure more open and make it easier for transcription factors to combine with other proteins, which in turn promotes gene expression. In recent years, three major families of HATs have been identified, including MYST family (MOZ, Ybf2, Sas2, TIP60), Gcn5-related N-acetyltransferase family (GNAT) and orphan family (CBP/EP300 and nuclear receptors) (17). HDACs can be classified into four categories: Class I HDACs, consisting of HDACs 1, 2, 3 and 8; Class II HDACs, consisting of HDACs 4, 5, 6, 7, 9 and 10; Class III HDACs, consisting of seven silencing regulatory proteins, including NAD-dependent protein deacetylases and ADP ribosylases, also known as Sirtuins; Class IV HDACs, containing only HDAC11, has sequence similarity compared to class I and II proteins (16). HATs relax chromatin structure and promote transcription through transferring the acetyl group from acetyl coenzyme A to histone amino acid terminus. In contrast, HDACs make the chromatin structure more compact by removing the acetyl group from the lysine terminus, thereby inhibiting transcription. Altered overall levels of histone acetylation have been shown to be connected with many tumor phenotypes (18). In general, hyperacetylation leads to increased gene expression, especially when proto-oncogenes are involved, and gene expression might be activated. Hypoacetylation of oncogenes is usually localized to the promoter simultaneously with DNA methylation, resulting in suppression of gene expression (19).

### 2.2 The Mechanisms of Histone Methylation

Histone methylation plays an important role in cell biological fate, such as DNA recombination and damage repair, gene expression and cell differentiation, and so forth (20). Histone methylation, catalyzed by histone methyltransferases (HMTs), occurs mainly on lysine and arginine residues located at histone tails. Arginine can be mono- or dimethylated, while for lysine, apart from mono- and dimethylated, it is able to be trimethylated (21). Histone methylation regulatory genes include protein arginine methyltransferases PRMTs, lysine methyltransferases KMTs (e.g. EZH2 and DOT1L), and histone demethylases HDMs (e.g. LSD1 and KDMs). The function of all KMTs is to bind s-adenosylmethionine (SAM) methyl donors to lysine methyl acceptors and facilitates methyl transfer from SAM to the telomeres (22). Protein arginine methylation reactions are mainly catalyzed by the protein arginine methyltransferase family, including PRMT1-9. These enzymes catalyze the reaction of transferring a methyl group from S-adenosylmethionine (AdoMet) to a guanidino nitrogen of arginine, which results in the formation of methylarginine and S-adenosylhomocysteine (AdoHcy) (23). Lysine methylation occurs mainly on histone H3 and H4, for example, six lysine residues, H3K4, H3K9, H3K27, H3K36, H3K79, and H4K20, are proven to be methylated in histones H3 and H4, respectively. Among them, acetylation can take place in histone H3 for H3K9 and H3K27. Lots of other lysine residues, H3K14, H3K18, H3K23 and H4K5, H4K8, H4K12 and H4K16, can only be acetylated rather than methylated. Methylation/acetylation in H3K9 and H3K27 performs different physiological functions, for instance, acetylation of H3K9 and H3K27 leads to gene transcriptional activation, whereas methylation of H3K9 and H3K27 represses transcription (14).

### 2.3 The Mechanisms of Histone Phosphorylation in Cancer

Histone phosphorylation occurs mainly at serine, tyrosine and threonine residues in the histone tails, which is regulated by various kinases and phosphatases, mediating biological fate such as DNA damage repair, chromatin remodeling, transcriptional activation and apoptosis (24). During mitosis, histone phosphorylation disrupts the balance of interactions between histones and DNA, resulting in an unstable chromatin structure, which ultimately allows the chromatin structure to remodel into homologous chromosomes (21). It is worth noting that histone phosphorylation mediates the transcriptional regulation of genes, particularly those that regulate the cell cycle and cell proliferation. For example, H3Ser10p and H2BSer32p are involved in epidermal growth factor (EGF)-mediated gene transcriptional activation (25, 26). In addition, H3ser10p mediates the transcriptional activation of proto-oncogenes such as c-myc and c-fos (26, 27). Histone H3 threonine 45 (H3T45) is phosphorylated with the participation of protein kinase C1, which promotes acetylation of H3K56 and ultimately regulates apoptosis and DNA replication (28, 29). A growing number of studies have shown that histone phosphorylation is involved in tumorigenesis and progression. For example, histone H4 phosphorylation is closely associated with liver regeneration and hepatocellular carcinoma (30). Increased levels of histone H3S10 phosphorylation is involved in the proliferation of gastric cancer cells and can be an independent prognostic indicator of vascular infiltration in gastric cancer (31, 32).

### 2.4 A Brief Description of Histone Ubiquitination

In post-transcriptional modifications, protein ubiquitination is a common process in cells (33, 34). Ubiquitination is a cascade reaction that relies on Adenosine Triphosphate (ATP) to link ubiquitin to a substrate protein. Ubiquitin activated by ubiquitin activating enzymes E1s is transferred to the ubiquitin binding enzymes E2s and ultimately transferred to the substrate protein by the ubiquitin ligases E3s (35). Among them, the ubiquitin ligases E3s are key enzymes in the ubiquitination process because of their specific recognition of the substrate protein (36). Histone ubiquitination is also involved in DNA damage repair, gene transcriptional regulation and genomic stability (37). Protein deubiquitination is catalyzed by deubiquitinating enzymes (DUB) and the dynamic balance of ubiquitination and deubiquitination plays an important role in cellular homeostasis. Therefore, their aberrant expression has been shown to be involved in the development and progression of many cancers (38–40).

### 2.5 A Novel Histone Modification: Sumoylation

Sumoylation has the same mechanism compared with ubiquitination, allowing for the covalent attachment of small ubiquitin-like modifiers (SUMO) to lysine residues of the substrate protein, which is a reversible post-transcriptional modification (41). Sumoylation acts as a negative regulator involved in the regulation of protein stability, gene transcription and ultimately repression of gene transcription (42, 43). Most of the substrate proteins for sumoylation are nuclear proteins. For example, the sumoylation of transcription factor can recruit chromatin-modifying enzymes to repress gene expression (44). In addition, sumoylation regulates the expression and activity of histone-modifying enzymes, suggesting a close relationship between sumoylation and epigenetic regulation (24). For instance, sumoylation of the transcription factor E2F1 promotes EZH2 transcription by increasing the binding of E2F1 to the EZH2 promoter (45). It has been shown that SUMO-1 is involved in the proliferation and apoptosis of endometrial cancer cells by increasing the level of histone H4 sumoylation (46). Another study shows that the sumoylation of transcription factor ETV1 promotes prostate cancer development (47), suggesting that sumoylation is involved in tumorigenesis and progression.

## 3 The Role of ncRNAs in GC

### 3.1 Overview of Noncoding RNAs in Cancer

Noncoding RNAs (ncRNAs) are a unique kind of RNA transcripts that are intensively recognized in eukaryotic genomes (48). Although over 75% of the human genome can be transcribed, among them, only *ca.* 2% of the transcription products are translated into proteins, and the remaining transcripts (which cannot encode proteins) are initially treated as junk transcription products (49). Transformed from junk transcription products to functional molecules that regulate cellular processes, ncRNAs regulate gene expression at different levels during physiological development, including signal transduction, chromatin remodeling, gene transcription and post-transcriptional modifications (50). Through their regulatory networks, ncRNAs can modulate many downstream targets to mediate specific cell biological fate (51). Growing evidence indicates that ncRNAs influence normal cell function and disease progression, especially in cancer development, which are considered as tumor suppressors and oncogenic factors (52, 53). Meanwhile, high-throughput sequencing technologies developed a lot, and increasing studies are showing that a little bit of ncRNAs have small open reading frames (sORFs), which can encode peptides or proteins (48). It has been demonstrated that HOXB-AS3, encoded by lncRNA, is shown to regulate tumor energy metabolism. FBXW7-185aa 18 and PINT-87aa 20, encoded by circRNA, can block tumor cell cycle and inhibit cell expansion. SHPRH-146aa 19, encoded by circRNA, can suppress tumor cell expansion and malignant phenotype. miPEP-200a and miPEP-200b, encoded by miRNAs, regulate epithelial-mesenchymal transition (EMT), leading to tumorigenesis and progression. According to transcription length, ncRNAs are mainly classified as long noncoding RNA (lncRNAs, linear, >200 nucleotides), circular RNA (circRNAs, covalently closed continuous loops), and short ncRNAs (linear, <200 nucleotides) (54).

### 3.2 A Brief Description of miRNAs and Their Role in GC

**Figure 1:**
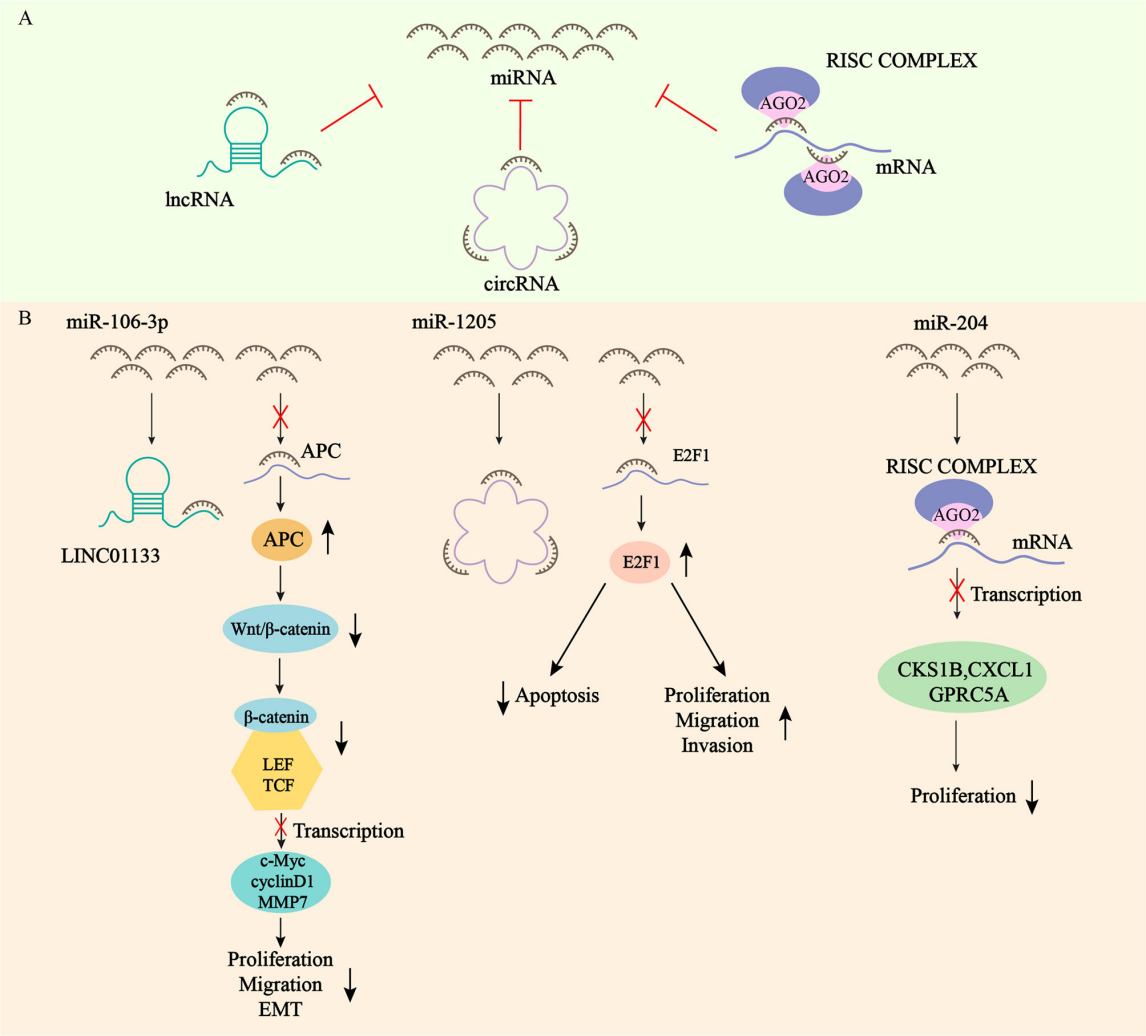
Network of competitive endogenous RNAs. **(A)** mRNAs, lncRNAs, and circRNAs all contain microRNA (miRNA) response elements (MREs) that can bind to miRNAs and regulate the expression of RNA targets at the post-transcriptional level. **(B)** LINC01133 exerts oncogenic effect by acting as a sponge for miR-106-3p, which regulates the expression of APC and inactivates the Wnt/β-catenin signaling pathway. circCYFIP2 exerts oncogenic effect by acting as a sponge for miR-1205, which regulates the expression of E2F1. miRNA-204 inhibits the transcription of CKS1B, CXCL1 and GPRC5A mRNAs by binding to AGO2 to form the RISC complex, which ultimately inhibits the proliferation of GC.

miRNAs are considered as a class of highly conserved tissue-specific small molecule non-protein-coding RNAs. Mature miRNAs are generated from primary miRNAs (pri-miRNAs) by two consecutive enzymatic reactions and loaded into RNA-induced silencing complexes (RISCs) containing Argonaute 2 (AGO2) (55, 56), which repress messenger RNAs (mRNAs) translation and promote mRNA degradation by directly combining with mRNA, and ultimately inhibit gene expression at the post-transcriptional stage to maintain intracellular homeostasis (57, 58). They are powerful regulatory factors of various biologically important activities such as cell growth and development (59). Regulatory mechanisms of miRNAs action include gene polymorphism, miRNA promoter methylation, interactions with RNA binding proteins (RBPs) or other RNAs, nucleotide modification of miRNA sequences at different stages of maturation, and unsymmetric miRNA strand selection (60) (36). miRNAs can repress the expression and activity of target RNAs at the post-transcriptional level, and the mechanism is the recognition of miRNA response elements (MREs) of target RNAs. MREs are usually located in the 3’ untranslated region (3’UTR) of the RNA, and can also be located in the 5’ untranslated region (5’UTR) and in the coding sequence (CDS). miRNAs recognize and bind sequence-complementary MREs at the 3’UTR of the target transcription product, and their inhibition of protein synthesis mechanism is not fully understood (56). When RNA transcripts have identical MREs on their 3’UTR, they can act as competing endogenous RNAs (ceRNAs), influencing each other’s expression by competing and combining with miRNAs from the same library. Each miRNA can target multiple RNAs, and at the same time, each RNA can be a target of multiple miRNAs. Ultimately, miRNAs can form a complex ceRNET with their associated transcripts (**Figure 1A**) (61). Herein, we highlight a few example from each category, with **Figure 1B** providing visual representations of the ceRNA network (62–64). Many physiological and pathological processes, including cardiovascular/metabolic diseases and cancers, are proven to be heavily dependent on miRNAs (65). Therefore, tumor development, such as cell proliferation, motility, apoptosis and angiogenesis, can be affected by aberrant expression of miRNAs (66). Recently, growing studies have demonstrated that deregulation of miRNAs expression is related to gastric cancer by acting as oncogenes and tumor suppressors, which can contribute to the diagnosis and treatment of GC (67–69). What’s more, there is growing evidence suggesting that circulating plasma and serum miRNAs can act as potential biomarkers for non-invasive tumor diagnosis, cancer therapy and prognosis (70–72).

### 3.3 The Mechanisms of lncRNAs in GC

lncRNA is defined as a transcript greater than 200 nt in length, and typically lacks the capability of encoding proteins (73). lncRNAs exert a variety of biological functions at the epigenetic level by regulating gene expression. As a result, tumorigenesis and progression are highly dependent on the dysfunctional lncRNAs (74). Recent studies disclose that a lot of lncRNAs are aberrantly regulated in GC, and altered lncRNA expression participates in cancer pathological processes, such as cell proliferation, metastasis, EMT, apoptosis, tumor stemness, drug resistance, etc. Therefore, it has the potential to act as a biomarker and therapeutic target for GC (75–78). Furthermore, it has been shown that the broad expression pattern in cancer, stability in circulating body fluids (plasma and urine) and tumor specificity of lncRNAs imply that subtype/tissue-specific expression of lncRNAs is important for the discovery of novel diagnostic, prognostic and therapeutic targets (79–81).

### 3.4 circRNAs: A Rising Star in GC Research Field

Circular RNAs (circRNAs) are covalent closed-loop structures formed by post-sniping of precursor mRNAs without 5’ caps or 3’ polyA tails, and they are also a class of endogenous RNAs without function of encoding proteins or peptides (82). Unlike linear RNA, circRNA has a special cyclic covalent bond structure, which makes them evolutionarily conserved and highly stable. Thus, they are inherently resistant to RNA exonucleases as well as RNaseR activity and are widely expressed in complex tissues and cell types at specific stages (83). Literature demonstrates that circRNAs have many biological functions, such as microRNA sponges, transcriptional regulation, regulation of mRNA stability, encoding functional proteins, and binding to RNA-binding proteins (84). Therefore, circRNAs have obvious impacts on many human diseases, such as diabetes, neurological/cardiovascular diseases, and cancers, thus exhibiting great potential as diagnostic and therapeutic targets (85, 86). In the case of GC, the importance of dysregulated circRNAs in gastric carcinogenesis, progression and clinicopathology has been demonstrated, thus showing the value of biomarkers for diagnosis and treatment (87, 88).

## 4 ncRNAs Regulate Histone Modification in GC

**Figure 2:**
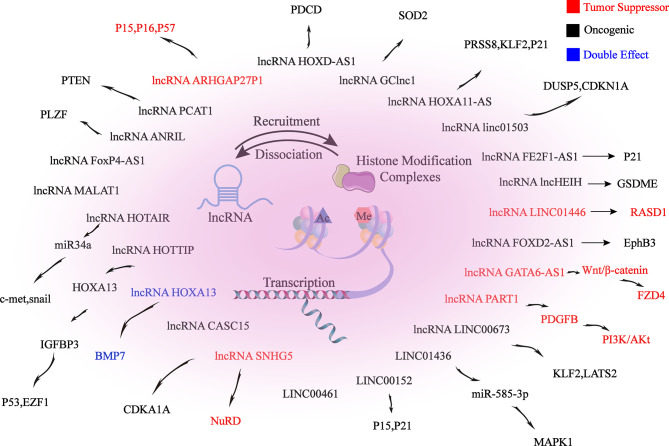
lncRNAs mediate histone modifications of target genes. lncRNAs induce changes in promoter histone modification patterns by recruiting histone modification complexes to target gene promoter-associated binding sites, thereby epigenetically regulating target gene expression, which ultimately alters the biological behaviors of GC.

**Figure 3:**
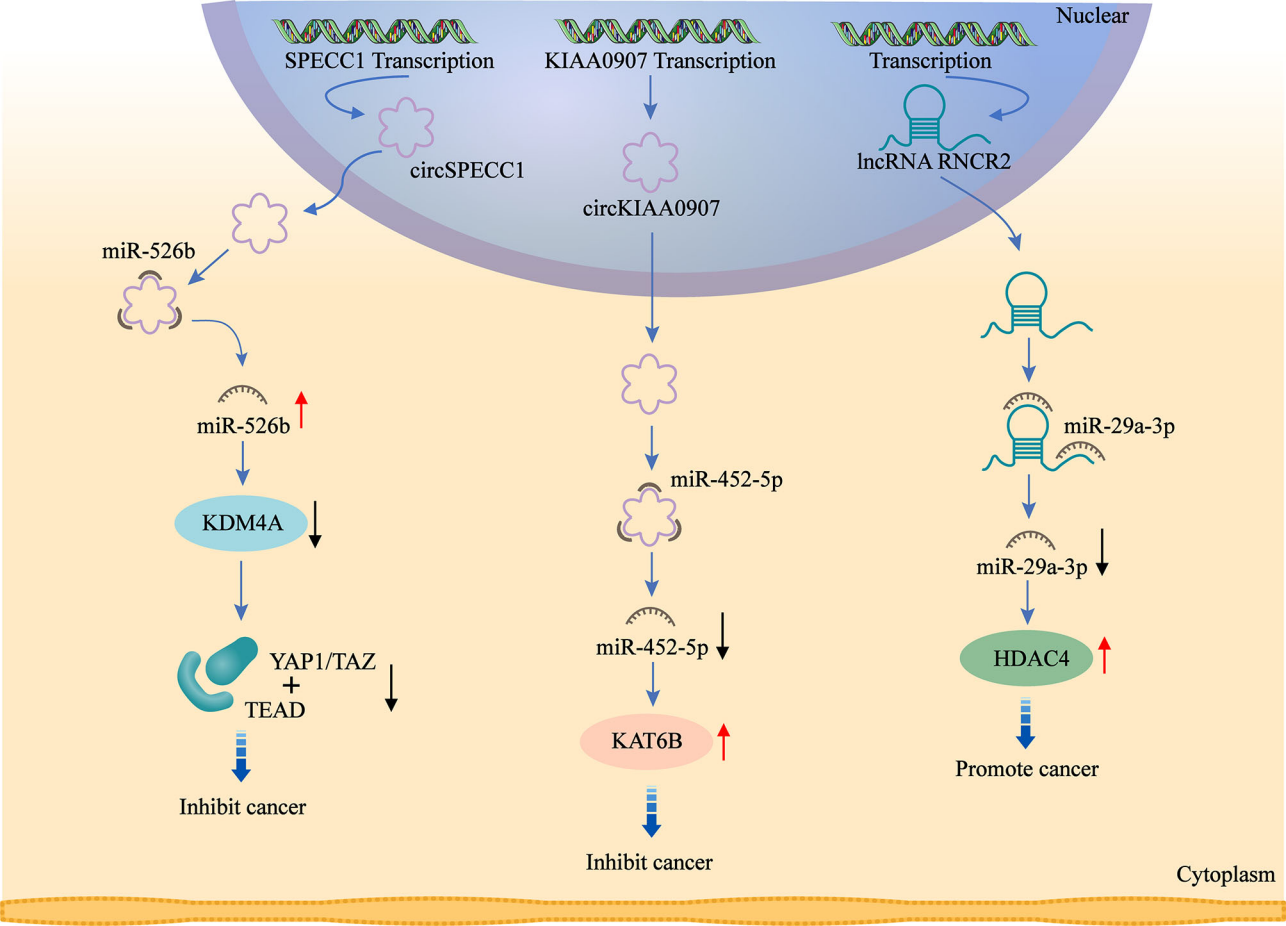
ceRNAs are involved in the regulation of histone modifiers. circ_0000745 acts as a sponge for miR-526b and promotes KDM4A expression. circKIAA0907 acts as a sponge of miR-452-5P and promotes KAT6B expression. lncRNA MIAT acts as a sponge for miR-29a-3p and promotes HDAC4 expression. ceRNAs alter histone and non-histone modifications by regulating the expression of histone modifiers, which affects downstream signaling targets and ultimately exerts their oncogenic or anti-tumor effects.

**Figure 4:**
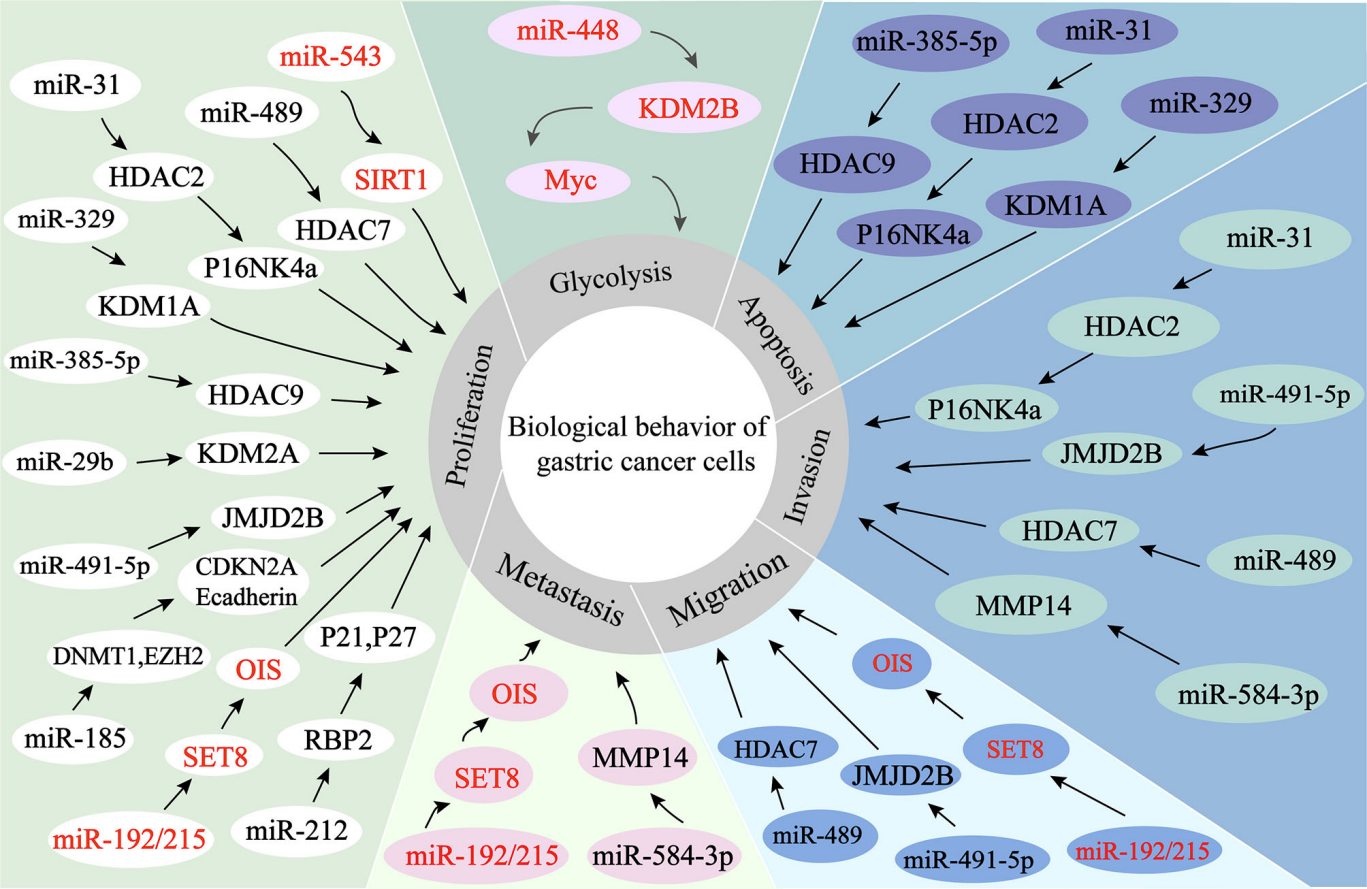
miRNAs regulate histone modification in GC. 1. miR-584-3P recruits EZH2 and EHMT2 to the MMP-14 promoter, thereby inhibiting the expression of MMP-14 and ultimately exerting its oncogenic effects. 2. miRNAs directly target histone modifiers and affect the alteration of histone or non-histone modifications patterns, ultimately leading to changes in the biological behaviors of gastric cancer cells.

Firstly, ncRNAs specify the histone modification pattern of target genes by recruiting tissue modification complexes directed to specific gene loci and might repress *cis* or *trans* transcription of target genes (**Figure 2**); Secondly, ncRNAs regulate target genes by participating in ceRNET as miRNA sponges (**Figure 3**); Thirdly, they can directly target histone modifiers and affect the alteration of histone or non-histone modifications pattern (**Figure 4**). Through these mechanisms, they drive the malignant biological behaviors of GC cells and ultimately promote tumor formation and progression (**Table 1** and **Table 2**).

**Table 1:** miRNAs regulate histone modification in GC.

miRNA	Expression	Target	Function	Reference
miR-584-3p	Downregulation	MMP-14 promoter	Tumor suppressor	(89)
miR-489	Downregulation	HKDC7	Tumor suppressor	(91)
miR-31	Downregulation	HDAC2	Tumor suppressor	(92)
miR-383-5p	Downregulation	HDAC9	Tumor suppressor	(93)
miR-329	Downregulation	KDM1A	Tumor suppressor	(94)
miR-212	Downregulation	RBP2	Tumor suppressor	(95)
miR-29b	Downregulation	KDM2A	Tumor suppressor	(96)
miR-491-5p	Downregulation	JMJD2B	Tumor suppressor	(97)
miR-185	Downregulation	DNMT1, EZH2	Tumor suppressor	(98)
miR-543	Upregulation	SIRT1	Oncogenic	(99)
miR-448	Upregulation	KDM2B	Oncogenic	(100)
miR-192/215	Upregulation	SET8	Oncogenic	(101)

**Table 2:** lncRNAs regulate histone modification in GC.

lncRNA	Expression	Mechanism	Target	Function	Reference
lncRNA SNHG5	Downregulation	Histone acetylation	MTA2, NuRD	Tumor suppressor	(102)
lncRNA MALAT1	Upregulation		EGFL7	Oncogenic	(103)
lncRNA MIAT	Upregulation		MiR-29a-3p, HDAC4	Oncogenic	(104)
lncRNA GATA6-AS1	Downregulation	Histone methylation	FZD4	Tumor suppressor	(105)
LINC01446	Downregulation		RASD1	Tumor suppressor	(106)
lncRNA PART1	Downregulation		PLZF, PDGFB	Tumor suppressor	(107)
lncRNA ARHGAP27P1	Downregulation		P15, P16, P57	Tumor suppressor	(108)
lncRNA HOXD-AS1	Upregulation		PDCD	Oncogenic	(109)
lncRNA HOXA11-AS	Upregulation		PRSS8, KLF2, P21	Oncogenic	(110)
lncRNA FEZF1-AS1	Upregulation		P21	Oncogenic	(111)
lncRNA linc01503	Upregulation		DUSP5, CDKN1A	Oncogenic	(112)
lncRNA FOXP4-AS1	Upregulation		EZH2, LSD1	Oncogenic	(108)
LINC00461	Upregulation		LSD1	Oncogenic	(113)
lncRNA PCAT1	Upregulation		PTEN	Oncogenic	(114)
lncRNA ANR1L	Upregulation		PLZF	Oncogenic	(115)
lncRNA HOTTIP	Upregulation		IGFBP3	Oncogenic	(116)
lncRNA HOTAIR	Upregulation		miR34a	Oncogenic	(117)
lncRNA HOXA13	Upregulation		BMP7	Oncogenic	(118)
lncRNA CASC15	Upregulation		CDKN1A	Oncogenic	(119)
LINC00673	Upregulation		KLF2, LAST2	Oncogenic	(120)
LINC01436	Upregulation		miR-585-3P	Oncogenic	(121)
lncRNA FOXD2-AS1	Upregulation		EphB3	Oncogenic	(122)
lncRNA lncHEIH	Upregulation		GSDME	Oncogenic	(123)
LINC00152	Upregulation		P15, P21	Oncogenic	(124)
lncRNA GClnc1	Upregulation	Histone acetylation, methylation	SOD2	Oncogenic	(125)

### 4.1 miRNAs Regulate Histone Modification in GC

#### 4.1.1 miRNAs Regulate the Expression of Target Genes by Recruiting Histone-Modifying Complexes

The expression of miR-584-3p is decreased in GC. miR-584-3p inhibits MMP-14 transcription by recruiting euchromatic histone lysine methyltransferase 2 (EHMT2) and enhancer of zeste homolog 2 (EZH2) to interact with AGO2, leading to enrichment of repressive epigenetic markers and reduced binding of YY1 to the MMP-14 promoter. YY1 (one member of the GLI-Krüppel protein family) directly targets the matrix metalloproteinase 14 (MMP-14) to promote MMP-14 expression in GC cells and tissues, resulting in tumorigenesis and invasion. miR-584-3p ultimately inhibits GC cell growth, metastasis and angiogenesis by suppressing the expression of MMP14 (90).

#### 4.1.2 miRNAs Regulate Histone Acetylation in GC

In GC cells, miR543 expression is upregulated, promoting cell cycle and proliferation, and thus positively correlates with the clinical phenotype of GC patients. By directly targeting the SIRT1 3 ‘-UTR, miR-543 can inhibit the expression of SIRT1 mRNA, thereby promoting GC cell proliferation and cell cycle progression. SIRT1, a class III histone deacetylase, is downregulated in GC and can inhibit gastric carcinogenesis and progression (90).

miR-489 is significantly downregulated in GC cells and tissues, and the downregulation can promote GC cell proliferation, invasion and migration, which is positively correlated with the prognosis of GC patients. Knockdown of HDAC7, a direct target of miR-489, can inhibit GC development as well as antagonize the effects of miR-489 inhibitors on GC cells. Taken together, by targeting HDAC7 and blocking the PI3K/AKT pathway, miR-489 exerts tumor suppressive effects in GC cell growth (99).

The overexpression of miR-31, which is aberrantly downregulated in GC cell lines, might inhibit proliferation and migration of GC cells, as well as induce apoptosis. miR-31 expression in GC is epigenetically regulated, and down-regulation of miR-31 is related to DNA methylation of promoter. In addition, HDAC2 can act as a direct target of miR-31 through combining with MREs in the 3’-UTR, and its expression is negatively regulated by miR-31. HDAC2 inactivation restores the activity of p16 ^INK4a^, which has anti-tumor effects in gastric cancer (91).

miR-383-5p expression is downregulated in GC cells and tissues. Therefore, miR-383-5p overexpression inhibits proliferation, induces apoptosis, as well as suppresses tumor growth in GC cell lines. HDAC9 is a direct target of miR-383-5p, which can be upregulated in GC cell lines. Therefore, it is closely associated with malignant development of GC. As a result, knockdown of HDAC9 in GC cell lines promotes apoptosis and inhibits proliferation, and microRNA-383-5p can suppress GC development through targeting HDAC9 expression (92).

#### 4.1.3 miRNAs Regulate Histone Methylation in GC

miRNA-329 expression is reduced in GC. miRNA-329 overexpression in GC cells promotes apoptosis and inhibits the malignant biological behaviors of GC cells. miRNA-329 mimics and siRNAs targeting KDM1A, a downstream target of miRNA-329, reduce the expression of KDM1A and increase the levels of H3K4me1 and H3K4me2. As a result, patient with high expression of miRNA-329 or low expression of KDM1A has a longer overall survival. In summary, microRNA-329 promotes apoptosis and inhibits proliferation, metastasis and growth by negatively regulating KDM1A in GC cells (93).

miR-448 is overexpressed in GC, promotes glycolysis and growth of GC, and is associated with poorer survival. The mechanism is that miR-448 expression is increased in GC, and the increasing expression of miR-448 upregulates the expression of the rate-limiting enzyme of glycolysis, Myc, through directly inhibiting KDM2B, which ultimately stimulates glycolysis (94).

miR-212 expression is aberrantly reduced in human GC cells and tissues. miR-212 is found to inhibit RBP2 expression through directly combining with the 3’UTR site of Retinoblastoma binding protein 2 (RBP2). RBP2, a newly identified histone demethylase, is overexpressed in GC. miR-212 exerts oncogenic effects in gastric cancer by suppressing RBP2 expression and increasing the expression of P21 ^CIP1^ and P27 ^kip1^ to arrest the cell cycle and inhibit cell colony formation (100).

miR-192/215 expression is significantly increased in GC cells and tissues. miR-192/215 can directly combine with the 3’UTR of SET8 to facilitate proliferation and metastasis of GC cells. Histone methyltransferase SET8 (KMT5A), one of the members of SET domain-containing methyltransferase family, is responsible for catalyzing monomethylation of H4K20me. And the expression of SET8 is downregulated in GC cells and tissues. Furthermore, in GC cells, SET8 triggers oncogene-induced senescence (OIS) and induces senescence through p53-dependent DNA damage induced oncogenes. Thus, by inhibiting the senescence signaling pathway, miR-192/215-SET8-p53 axis exacerbates GC development (95).

RUNX3 is a transcription factor that binds directly to the region of miR-29b promoter and cooperates with Smad3, leading to higher activity of miR-29b promoter. Both miR-29b and RUNX3 are decreased in GC tissues, with positively correlated expression. miR-29b directly binds to KDM2A 3′-UTR and negatively regulates the expression of KDM2A, serving as a target of miR-29b. Through this mechanism, the proliferation and migration of GC cells are inhibited (101).

As miR-491-5p is decreased in GC cells, tissues and serum, its high expression can inhibit proliferation and invasion in GC. By targeting JMJD2B (KDM4B), miRNA-491-5p can inhibit malignant biological behaviors in GC cell lines, exhibiting the potential as a gastric cancer-associated marker (96).

#### 4.1.4 miRNAs Regulate Histone Acetylation and Methylation in GC

The GKN1-miR-185-DNMT1 axis exerts oncogenic effects in gastric cancer through mediating epigenetic alterations and cell cycle. Gastrokine 1 (GKN1) reduces GC cell proliferation, viability, and colony formation, as well as blocks cell cycle. The mechanisms are as follows: 1. GKN1 inhibits DNMT1 and EZH2 expression through upregulation of miR-185 expression. miR-185 negatively regulates the inhibitory actions of GKN1 on tumorigenicity, expression of DNMT1 and EZH2, as well as DNMT1 activity; 2. GKN1-induced miR-185 leads to CDKN2A and E-cadherin re-expression through demethylation of CDKN2A and E-cadherin promoter regions; 3. GKN1 negatively regulates DNMT1 through repressing HDAC1 and inducing Tip60 in a miR-185-free manner (97).

### 4.2 lncRNAs Regulate Histone Modification in GC

#### 4.2.1 lncRNAs Regulate Histone Acetylation in GC

lncRNA SNHG5 is a transcript consisting of six exons of U50HG, which has been reported to be the host gene of snoRNAs U50 and U50’. SNHG5 expression is aberrantly decreased in GC cells and tissues, inhibiting metastasis and proliferation in GC cells. The NuRD complex participates in chromatin remodeling and histone deacetylase with subunits, including MTA1, MTA2, HDAC1 and HDAC2. The mechanism is that the overexpression of SNHG5 affects the formation of the NuRD complex by blocking the transference of MTA2 from the cytoplasm to the nucleus, resulting in significant upregulation of histone H3 and non-histone p53 acetylation levels, which interferes with nucleosome remodeling and the formation of the histone deacetylation complex. As a result, the metastasis and growth of GC cells are suppressed (98).

The expression of lncRNA MALAT1 is aberrantly upregulated in GC cells and tissues. MALAT1 induces EGFL7 protein expression by altering H3 histone acetylation at the region of EGFL7 promoter. Epidermal growth factor-like domain-containing protein 7 (EGFL7), also known as vascular endothelial statin, is encoded by the EGFL7 gene and involved in regulating the formation of vascular ducts and the progression of many cancers. MALAT1 stimulates migration and invasion of GC cell lines through the above mechanism (102).

MIAT expression is significantly increased in GC. Knockdown of lncMIAT inhibits the malignant biological behaviors of GC cells. lncMIAT competitively combines with miR-29a-3p through serving as a sponge for miR-29a-3p, thereby increasing HDAC4 expression and ultimately exerting an oncogenic effect in GC (103).

#### 4.2.2 lncRNAs Regulate Histone Methylation in GC

lncRNA HOXA11-AS is specifically increased in GC, promoting GC cell proliferation, migration and invasion, with apoptosis inhibition. HOXA11-AS is thought to bind directly to LSD1, EZH2, WDR5, STAU1, DNMT1 and AGO2 in tumor cells, acting as a frame for EZH2 and LSD1/DNMT1 to downregulate PRSS8, KLF2 and P21 expression, thus promoting GC. EZH2 can recognize and bind to PRSS8, KLF2 and P21 promoters, which in turn drives H3K27me3 modification. While LSD1 contributes to H3K4 demethylation by binding directly to the PRSS8 promoter. Moreover, HOXA11-AS can also serve as a miR-1297 sponge by binding to miR-1297, releasing repressive effect on EZH2 mRNA. In addition, EZH2 is further recruited by HOXA11-AS to epigenetically inhibit the expression of KLF2 and PRSS8 (104, 109).

lncRNA FEZF1-AS1 is located at chromosome 7, which is a transcript of 2564 bp in length. The transcription factor SP1 specifically recognizes and binds to the FEZF1-AS1 promoter binding site, increasing the expression of FEZF1-AS1 in GC. GC cells are proliferated through the following mechanisms: Firstly, FEZF1-AS1 causes G1-S cell cycle arrest; Secondly, FEZF1-AS1 binds to LSD1, causing p21 promoter H3K4me2 demethylation and inhibiting the expression of the downstream target p21 (110).

In GC, Linc01503 is regulated by the transcription factor 1 (EGR1) and its expression is upregulated. Linc01503 epigenetically represses the expression of cyclin-dependent kinase inhibitor 1A (CDKN1A) and dual-specificity phosphatase 5 (DUSP5) by recruiting LSD1 and EZH2. EZH2 binds to CDKN1A and DUSP5 promoters, driving histone 3 lysine 4 trimethylation (H3K4me3). While LSD1 binds to DUSP5 and CDKN1A promoters, mediating H3K4me2 demethylation. Ultimately, GC cell cycle progression and proliferation can be promoted by Linc01503, with apoptosis inhibited (111).

The expression of HOXD-AS1 is increased in DDP-resistant GC patients. DDP sensitivity of GC cells can be promoted by knockdown of HOXD-AS1, which can promote cisplatin resistance in GC through recruiting EZH2 and upregulating H3K27me3 levels on the region of PDCD4 promoter in GC cells to epigenetically silence PDCD4 (104).

The expression of lncRNA GATA6 antisense RNA 1 (GATA6-AS1) was decreased in GC cells, which promotes the malignant development of GC cells, such as proliferation, metastasis and invasion. Its overexpression can inhibit tumor growth as well as lymph node metastasis (LNM) in GC. GATA6-AS1 can downregulate the expression of FZD4 by specifically recruiting and binding with EZH2 to increase the enrichment of H3K27me3 in the region FZD4 promoter, which in turn inhibits the Wnt/β-catemin downstream pathway, ultimately leading to the blockage of GC progression (112).

lncRNA 01446 (LINC01446), a 3484-bp ncRNA, is located at chromosome 7p12.1. LINC01446 is downregulated in GC, which is correlates with metastasis and poor prognosis in GC patients. This lncRNA can epigenetically repress Ras-related dexamethasone inducible 1 (RASD1) by recruiting LSD1 to the region of RASD1 promoter, ultimately promoting malignant behaviors of tumor cells (105).

lncRNA PART1 is locate at chromosome 5q12.1. PART1 is decreased in tumor cells and tissues, which is closely related to poor prognosis of GC patients. PART1 overexpression inhibits invasion and metastasis of GC cells. Mechanistically, PART1 acts as a role of tumor suppressor by interacting with androgen receptor (AR) and stimulating the PLZF gene expression in GC cells. Then, co-occupancy of EZH2 and PLZF on the PDGFB promoter region enable histone 3 lysine 27 trimethylation (H3K27me3) to epigenetically silence PDGFB and suppress the PI3K/Akt pathway (106).

Rho GTPase-activating protein (ARHGAP) is a family of Rho homologous GTPase activating proteins that exert oncogenic effects by aberrant regulation of Rho/Rac/Cdc42-like GTPases (107). ARHGAP20 gene is confirmed to be located at chromosome 17q24.1, which is decreased in GC cells, tissues and plasma. Low expression of ARHGAP27P1 is related to adverse clinical features, suggesting a poor prognosis for GC patients. Mechanistic investigations showed that ARHGAP27P1 inhibits proliferation and blocks the cell cycle through combining with Jumonji-domain containing 3 (JMJD3), leading to a significant downregulation of H3K27me3 levels, which ultimately epigenetically induces transcription of p15, p16 and p57 genes (126). Highly expressed FOXP4-AS1 can promote proliferation, migration as well as metastasis of GC cells by interacting with EZH2/LSD1 (127).

LINC00461 is upregulated in GC tissues, which is positively correlated to TNM staging and lymphatic metastasis, mediating cell proliferation and apoptosis in GC through interacting with LSD1, thereby aggravating the progression of GC (108).

lncRNA prostate cancer–associated transcript 1 (PCAT‐1) is upregulated in CDDP‐resistant GC cell lines and tissues. Mechanistically, PCAT-1 epigenetically silences PTEN gene by recruiting EZH2 to the region of PTEN promoter, resulting in H3K27 trimethylation in the PTEN gene promoter region, ultimately leading to the progress of cisplatin resistance in GC cells. Trough inhibiting of PCAT-1 and upregulating PTEN expression, cisplatin sensitivity of CDDP‐resistant GC cells can be enhanced (113).

Tumor suppressor PLZF inhibits proliferation, metastasis and invasion of GC cells. The expression of PLZF is downregulated and negatively correlates with the expression level of lncRNA ANRIL in GC. This lncRNA recruits EZH2, a core member of PRC2, in turn catalyzes H3K27 trimethylation, which then drives PLZF gene silencing by increasing DNA methylation of PLZF. In summary, lncRNA ANRIL suppresses EMT as well as cell proliferation through epigenetic regulation of the tumor suppressor PLZF (114).

lncRNA HOTTIP causes hypomethylation of the HoxA13 promoter E1 site and elevates levels of H3K4me3 by decreasing the recruitment of DNA methyltransferases DNMT1 and DNMT3b at the HoxA13 promoter E1 site, increasing the binding of WDR5 and MLL complexes, which ultimately epigenetically activates HoxA13 expression. Meanwhile, IGFBP3 and HOTTIP are shown to be downstream targets of HoxA13 in GC cells. The expression of HoxA13 and HOTTIP shows positive feedback. HoxA13 trans-activated the expression of IGFBP-3 gene by binding to the Hox binding element at the IGFBP-3 promoter region. IGFBP-3 has been reported to inhibit migration, invasion and EMT in GC through inhibiting invasion factors, such as MMP14 and urokinase-type fibrinogen activator (115). However, IGFBP-3 might enhance division and metastasis in GC CS12 cell line, and knockdown of IGFBP-3 inhibits the poor biological behaviors of GC cells (128).

lncRNA HOTAIR is highly upregulated in GC, which promotes metastasis and invasion of GC cells by inducing EMT, and its overexpression predicts poor prognosis. HOTAIR epigenetically represses miR34a and activates the expression of its downstream targets C-Met (HGF/C-Met/Snail pathway) and Snail by recruiting the multiple sparing repressor complex 2 (PRC2, including EZH2, SUZ12, etc.) to bind directly to the region of miR34a promoter and induce H3K27 trimethylation. Thereby GC cell EMT process and tumor metastasis are accelerated (116).

Another study shows that the expression level of the epithelial marker E-cadherin (CDH1) is increased in HOTAIR knockdown GC. By contrast, the expression levels of mesenchymal markers are downregulated in HOTAIR knockdown GC cells, such as Snail, Slug, Twist, N-cadherin and β-catenin. HOTAIR knockdown derepresses the histone marker H3K27me3 for E-cadherin and induces conversion of the E-cadherin promoter to the active marker acetylated H3K27. In addition, the loss of SUZ12 with the involvement of the active acetyltransferase CBP leads to an increase in acetylation of H3K27 (129). In summary, in the region of E-cadherin promoter, HOTAIR can epigenetically repress E-cadherin by turning acetylation of histone H3 lysine 27 into methylation, which ultimately promotes EMT of GC (130).

Reprogramming of cancer cells into induced pluripotent stem cells (iPSCs) might be a novel method to suppress tumorigenesis. Another research reported the successful reprogramming of the human gastric cell lines (CS12s) into induced pluripotent stem cell–like cell lines (CS12iPSLCs) by utilizing Jun dimerization protein 2 (JDP2) and octamer-binding protein 4 (OCT4). The study shows that CS12iPSLCs exhibit reduced tumorigenicity *in vivo* and reduce colony formation, proliferation, invasion, migration, and drug resistance *in vitro*. The oncogenic function of Bone morphogenetic protein 7 (BMP7) is switched by lncRNA HOXA13 axis and loses in CS12iPSLCs. H3K4me3 can be increased by recruiting HOXA13, MLL1, WDR5 and HOTTIP at the BMP7 promoter S1 site, thereby activating the expression of BMP7 and promoting the process of GC in CS12. H3K27me3 can be enhanced by recruiting HOXA13, ZEH2, JARID2 and HOTAIR at the BMP7 promoter S2 site, thereby epigenetically suppressing BMP7 expression and inhibiting the progression of GC in CS12iPSLC. In summary, the HOXA13-HOTAIR and HOXA13-HOTTIP axis are recruited to different sites of the BMP7 promoter, leading to different outcomes of GC (117).

lncRNA CASC15 (cancer susceptibility candidate 15) is located at chromosome 6p22.3, which regulates GC cell biological behaviors and is involved in tumorigenesis and progression. The mechanisms are as follows: Firstly, CASC15 regulates GC cell growth partially by serving as a bridge between EZH2 and WDR5 in nucleus, epigenetically silencing CDKN1A. Moreover, in cytoplasm, CASC15 mediates GC cell migration in part by serving as a ceRNA for miR-33a-5p, which competitively binds to ZEB1 (118).

lncRNA LINC00673, an intergenic lncRNA, is located at chromosome 17q25.1. The expression of LINC00673 is aberrantly increased in GC cells. Knockdown of LINC00673 suppresses invasion as well as proliferation, with promoted apoptosis. LINC00673, activated by transcription factor SP1, exerts its oncogenic effects partly through serving as a frame for EZH2 and LSD1 to epigenetically suppress KLF2 and LATS2 genes, promoting the progression of GC (119).

LINC01436 is highly expressed in gastric cancer tissues and cells. High LINC01436 level is associated with poor patient prognosis. In addition, knockdown of LINC01436 inhibits migration and proliferation, with apoptosis promoted. Mechanistically, LINC01436 mediates trimethylation of H3K27 at the miR-585-3p promoter through enhancing EZH2, epigenetically silencing miR-585-3p expression, which in turn upregulates mitogen-activated protein kinase 1 (MAPK1) expression, ultimately promoting GC progression (120).

lncRNA FOXD2-AS1 expression is significantly increased in GC and positively related to the tumor size, later pathological stage as well as poor prognosis. Overexpression of FOXD2-AS1 can accelerate tumor development and predict poor prognosis in GC patients by EZH2 binding to the region of EphB3 promoter and inducing H3K27me3 modification, or LSD1 combining with the region of EphB3 promoter and inducing demethylation of H3K4, ultimately epigenetically silencing of EphB3 (121).

Overexpression of lncHEIH in GC promotes proliferation, migration, tumorigenesis, and expansion of GC stem cells, leading to tumor malignant transformation. lncHEIH can upregulate EZH2 expression, while recruiting and binding EZH2 in the GSDME promoter region and inducing H3K27me3 modification in this region to epigenetically silence GSDME expression, thereby promoting GC progression (122).

lncRNAs 152 (LINC00152) expression is significantly increased in gastric cancer cells and tissues, which is correlated with lymph node metastasis, depth of tumor invasion, higher TNM stage as well as poor prognosis. LINC00152 epigenetically silences the expression of p15 and p21 through combining with EZH2, ultimately accelerating cell cycle progression and proliferation in GC (123).

#### 4.2.3 lncRNAs Regulate Histone Acetylation and Methylation in GC

lncRNA BC041951, named gastric cancer-associated lncRNA 1 (GClnc1), is upregulated in GC and closely associated with poor prognosis of patients. Mechanistically, lncRNA GClnc1 might serve as a modular scaffold to recruit WD repeat protein 5 (WDR5) and histone acetyltransferase KAT2A to the region of the superoxide dismutase 2 mitochondria (SOD2) gene promoter (one of the target genes of the WDR5/KAT2A complex), increasing the levels of H3K4 trimethylation and H3K9 acetylation in the promoter region of SOD2, thereby epigenetically promoting the expression of SOD2. As a result, GC cell proliferation, invasion, tumor growth can be promoted, in addition, chemotherapy resistance can be controlled (124).

### 4.3 circRNAs Regulate Histone Modification in GC

#### 4.3.1 circRNAs Regulate Histone Acetylation in GC

circMRPS35 suppresses GC development through recruiting KAT7 to mediate histone modification. This study shows that circMRPS35 expression is significantly decreased in GC and related to the clinicopathological features and better prognosis of patients. circMRPS35 exerts oncogenic effect by inhibiting invasion and proliferation of GC cells *in vitro* and *in vivo*. The mechanism is that circMRPS35 enriches H4K5 acetylation at the regions of FOXO1 and FOXO3a promoters by recruiting the histone acetyltransferase KAT7, which ultimately epigenetically activates the expression of FOXO1 and FOXO3a, and induces subsequent responses in their downstream targets, including upregulation of p21, p27 and E-calmodulin, with downregulation of Twist1 expression, ultimately inhibiting cell proliferation and invasion (125).

Another study showed that circKIAA0907 is downregulated in GC. Upregulation of circKIAA0907 induces cell cycle arrest, proliferation and apoptosis, as well as inhibits autophagy. Mechanistically, circKIAA0907 serves as a sponge of miR-452-5p to increase lysine acetyltransferase 6B (KAT6B) expression in GC. KAT6B is a target gene of miR-452-5p, which has been found to play an regulatory role in many cancers. Shi et al. demonstrates that microRNA-4513 induces EMT and cell proliferation by targeting KAT6B (131). circKIAA0907 inhibits GC development and progression through downregulating miR-452-5p and upregulating KAT6B. This study demonstrates the potential diagnostic and therapeutic value of circKIAA0907, which can serve as a tumor suppressor in GC *via* the miR-452-5p/KAT6B axis (132).

#### 4.3.2 circRNAs Regulate Histone Methylation in GC

circ_0000745 (circ_SPECC1), transcribed from the SPECC1 gene, is downregulated in GC tissues and blood. It exerts anti-tumor effects by significantly inhibiting metastasis and proliferation of GC cells, with apoptosis promoted. circ_SPECC1 is shown to bind directly to miR-526b, which in turn affects miR-526b downstream signaling targets. miR-526b is downregulated in GC cells and tissues, inhibiting invasion and proliferation, with apoptosis promoted. Lysine demethylase 4A (KDM4A, also known as JMJD2A), a member of the histone demethylase family, is also an important target of miR-526b, whose expression is shown to be increased in various cancers. Huang et al. (133) demonstrates that KDM4A expression is aberrantly increased in GC tissues and correlated with clinicopathological features. Inhibition of KDM4A expression weakens transformation and growth of GC cells, with apoptosis promoted. In GC, YAP1/TAZ serve as downstream target genes of KDM4A, promoting transcription through binding to the transcription factor TEAD, ultimately leading to tumor formation (134). In summary, circ_SPECC1 can improve the inhibiting effect of miR-526b at the downstream signaling target YAP1/KDM4A, with invasion and growth of GC cells inhibited (135).

## 5 ncRNAs Are Regulated by Histone Modification in GC

Altering the ncRNA histone modification pattern by recruiting histone modifiers into the ncRNA promoter epigenetically regulates ncRNA expression, which in turn changes downstream signaling pathways and target genes. This mechanism ultimately plays an essential role in GC (**Table 3**).

**Table 3:** ncRNAs regulated by histone modification in EC.

ncRNA	Expression	Mechanism	Target	Function	Reference
miR-142-5p	Upregulation	Histone demethylation	CD9	Oncogenic	(136)
miR-155	Upregulation	Histone acetylation	SOX1	Oncogenic	(137)
miR-454	Upregulation	Histone deacetylation	CHD5	Oncogenic	(138)
miR-34a	Downregulation	Histone deacetylation	CD44	Tumor suppressor	(139, 140)
miR-330-3p	Downregulation	Histone deacetylation	MSI1	Tumor suppressor	(141)
miR-375	Downregulation	Histone deacetylation	YAP1, TEAD4, CTGF	Tumor suppressor	(142)
miR-454	Upregulation	Histone deacetylation	CHD5	Oncogenic	(138)
miR-133	Downregulation	Histone deacetylation,	mcl-1, Bcl-xL	Tumor suppressor	(143)
HOXC-AS3	Upregulation	Histone methylation, Histone acetylation	MMP7, WNT10B, HDAC5	Oncogenic	(144)
lncRNA FENDRR	Downregulation	Histone deacetylation	FN1, MMP2, MMP9	Tumor suppressor	(145)
lncRNA HRCEG	Downregulation	Histone deacetylation	–	Tumor suppressor	(145)

### 5.1 miRNAs Are Regulated by Histone Modification in GC

#### 5.1.1 miRNAs Are Regulated by Histone Acetylation in GC

miR-155 expression is increased in GC and exhibits pro-carcinogenic effects. MRTF-A (MKL1) promotes miR-155 expression by inducing the acetylation of histones and the recruitment of RNA polymerase II in the region of miR-155 promoter through the Wnt-β-catenin pathway. MRTF-A can act as a coactivator of serum response factor (SRF), participating in cell apoptosis, differentiation, proliferation and migration. miR-155 inhibits SOX1 expression through combining with the 3’UTR of SOX1, promoting migration of tumor cells. In summary, MRTF-A/miR-155/SOX1 axis can mediate metastasis of GC (146).

miR-34a and miR-34b/c, derived from miR-34 family, participate in cell cycle arrest, senescence and apoptosis in tumor. Mechanistically, Sirt7 can epigenetically inhibit miR-34a expression by deacetylating H3K18ac, ultimately preventing apoptosis in gastric cancer cells. The expression of Sirt7, a family member of NAD^+^-dependent protein deacetylases, is significantly upregulated in GC, and its knockdown promotes apoptosis and reduces the growth of tumor (137).

miR-330-3p expression is downregulated in GC. However, the ectopic expression of miR-330-3p decreases migration, proliferation, colony formation, and EMT in tumor cells. Treatment of GC cells with the histone deacetylase inhibitor trichostatin A (TSA) and the DNA methylation inhibitor 5-aza-CdR (AZA) enhances the expression of miR-330-3p. This indicates that the downregulation of miR-330-3p is partially mediated through hypermethylation in the region of the gene promoter. In addition, MSI1 is a target gene of miR-330-3p in GC cells. MSI1, an RNA binding protein (RBP), is upregulated in GC cell lines and tissues through combining with the mRNA 3’UTR sequences, leading to translational repression. Moreover, its expression is negatively related to the expression of miR-330-3p in GC tissues (139).

miR-375 expression is downregulated in GC due to methylation of its promoter and histone deacetylation, which is related to poor prognosis and lymph node metastasis. While miR-375 ectopic expression inhibits cell proliferation and tumor growth. miR-375 specifically and directly inhibits the expression of YAP1, TEAD4 and CTGF through combining with their 3’UTRs. YAP1, a co-activator of transcription, is a crucial downstream signaling molecule of the Hippo pathway. It mainly combines with the transcription factor TEAD and exerts oncogenic effects. The expression of CTGF is mediated by YAP1 and TEAD in GC cells. Overall, miR-375 is downregulated in GC cells due to its promoter methylation, which results in the co-activation of YAP1/TEADs-CTGF in the Hippo signaling pathway to promote gastric carcinogenesis (141).

Histone deacetylase 3 (HDAC3) is significantly upregulated in GC tissues and cells. However, knockdown of HDAC3 inhibits the growth of GC cells. HDAC3 overexpression upregulates miR-454 expression, which is significantly related to malignant clinical features in GC patients, implying that it can act as a biomarker for poor prognosis. Moreover, CHD5 is suggested to be a target of miR-454. The ectopic expression of CHD5 inhibits cell proliferation and tumorigenicity, as well as leads to cellular senescence. CHD5 expression is downregulated in GC and negatively associated with miR-454 levels. In summary, HDAC3 targets CHD5 through miR-454 to regulate the growth of GC cells (142).

Histone modification of the miR-133 family at the promoter region leads to a significant decrease of miR-133 expression in GC. Restoration of miR-133b/a-3p expression can target the anti-apoptotic molecules Bcl-xL and Mcl-1 to inhibit proliferation and induce apoptosis in GC cells, thereby suppressing GC growth. In summary, miR-133b/a-3p is regulated by histone modifications, exerting oncogenic effects in GC through targeting Bcl-xL and Mcl-1 (138).

Histone deacetylase 1 (HDAC1) knockdown inhibits cell metastasis and adhesion in GC cells through upregulating miR-34a. The mechanism is that the HDAC1/miR-34a axis regulates CD44 expression, activation as well as its downstream factors, including LIM domain kinase 1 (LIMK-1), matrix metalloproteinase (MMP)-2, ras homolog family member A (RhoA), and Bcl-2. The former three proteins participate in the organization of the microtubulin and actin cytoskeletons as well as the formation of cellular pseudopods. Thus, the depletion of HDAC1 can decreases the metastatic ability of GC cells through the miR-34a/CD44 axis (143).

#### 5.1.2 miRNAs Are Regulated by Histone Methylation in GC

LSD1 is thought to be a demethylase of H3K4me1/2, H3K9me1/2 and several non-histone proteins, which is extensively expressed in GC tissues. LSD1 deletion leads to upregulation of the migration inhibitory factor CD9 through decreasing intracellular miR-142-5p expression, which ultimately inhibits GC migration (140).

### 5.2 lncRNAs Are Regulated by Histone Acetylation in GC

lncRNA HOXC-AS3, located at chromosome 12q13.13, is defined as an antisense transcript of HOXC10. Gaining of H3K27ac and H3K4me3 activation of the promoter contributes to driving HOXC-AS3 gene in GC cells and tissues. By binding to YBX1, HOXC-AS3 transcriptionally regulates genes, such as MMP7, WNT10B and HDAC5. They are related to the biological behaviors of GC cells, ultimately inducing migration and proliferation in GC. In addition, HDAC5 gene is a member of the histone deacetylase (HDAC) family. Therefore, this study suggests that HOXC-AS3 is regulated by histone modifications, which in turn regulates HDAC5 to promote GC (136).

The lncRNA FENDRR is a gene of length 3099nts and located at Chr3q13.31. The expression of FENDRR is reduced in GC, which is correlated with poor prognosis and clinicopathological characteristics in GC patients. The histone deacetylase 3 (HDAC3) drives metastasis of GC cells through epigenetic silencing of FENDRR, induction of fibronectin1 (FN1) expression, and activation of MMP2/MMP9 (144).

lncRNA HRCEG is decreased in GC cells, which is an HDAC1-regulated RNA affecting GC cell proliferation and EMT transformation. HRCEG expression is negatively regulated by histone deacetylase 1 (HDAC1). The overexpression of HRCEG inhibits GC cells proliferation and EMT process (145).

## 6 Conclusions and Future Perspectives

Recently, growing evidence indicates that epigenetic alteration is a critical factor involved in tumorigenesis and progression. Research progress has been achieved in the use of epigenetic methods to treat cancer. For instance, numerous drugs targeting epigenetic pathways have achieved clinical efficacy, including inhibitors of DNMTs and HDACs. However, the mechanisms of epigenetic alterations in tumorigenesis and progression have not been fully explored. In this review, we attempt to comprehensively describe the important role of ncRNA and histone modification interactions in the pathogenesis of GC, thus providing a solid basis for identifying more specific and effective epigenetic therapeutic agonists/inhibitors. As the field of ncRNAs continues to evolve, there are many technologies that support the feasibility of ncRNA-based therapies. For ncRNAs with down-regulated expression, their expression levels can be enhanced by liposomes/nanoparticles from virus- or plasmid-based expression vectors. For ncRNAs with up-regulated expression, RNAi/shRNAs can be constructed or CRISPR-Cas9 can be used to suppress their expression levels. However, efforts are still needed in ncRNA-based therapy for tumors. Moreover, considering the limited efficacy of monotherapies and the possibility of serious adverse effects, we should achieve more progress in the field of epigenetic drugs combined with other anti-tumor drugs for cancer treatment, which will be a boon for GC patients.

## Author Contributions

QY and YD were responsible for the review of the literature. QY and YC wrote the manuscript. YC and RG edited the manuscript. QY and YD drew the tables and pictures. TY, QW, and ZX designed the study and made valuable discussions and revisions to the manuscript. All authors contributed to the article and approved the submitted version.

## Conflict of Interest

The authors declare that the research was conducted in the absence of any commercial or financial relationships that could be construed as a potential conflict of interest.

## Publisher’s Note

All claims expressed in this article are solely those of the authors and do not necessarily represent those of their affiliated organizations, or those of the publisher, the editors and the reviewers. Any product that may be evaluated in this article, or claim that may be made by its manufacturer, is not guaranteed or endorsed by the publisher.
